# Worldwide Prevalence of Small Ruminant Lentiviruses in Sheep: A Systematic Review and Meta-Analysis

**DOI:** 10.3390/ani11030784

**Published:** 2021-03-11

**Authors:** Ricardo de Miguel, Marta Arrieta, Ana Rodríguez-Largo, Irache Echeverría, Raúl Resendiz, Estela Pérez, Héctor Ruiz, Marta Pérez, Damián de Andrés, Ramsés Reina, Ignacio de Blas, Lluís Luján

**Affiliations:** 1 Department of Animal Pathology, University of Zaragoza, 50013 Zaragoza, Spain; ricardodemiguel@unizar.es (R.d.M.); martaa19957@gmail.com (M.A.); anarlg@unizar.es (A.R.-L.); rulirepo@gmail.com (R.R.); eperez@unizar.es (E.P.); hectorruiz353@gmail.com (H.R.); deblas@unizar.es (I.d.B.); 2 Institute of Agrobiotechnology, CSIC-Government of Navarra, 31192 Mutilva, Spain; irache.echeverria@unavarra.es (I.E.); damian.deandres@csic.es (D.d.A.); ramses.reina@unavarra.es (R.R.); 3 Department of Anatomy, Embriology and Genetics, University of Zaragoza, 50013 Zaragoza, Spain; mmperez@unizar.es; 4 Instituto Universitario de Investigación Mixto Agroalimentario de Aragón, University of Zaragoza, 50013 Zaragoza, Spain

**Keywords:** small ruminant lentiviruses, meta-analysis, prevalence, maedi-visna, seroprevalence

## Abstract

**Simple Summary:**

Maedi-visna is a progressive wasting disease of sheep that leads to decreased animal condition and production. It is caused by Small Ruminant Lentiviruses (SRLV), single-stranded RNA viruses with high mutational potential. There is neither treatment nor vaccine against SRLV and proper diagnosis is the key element for efficient control measures against positive animals. This systematic review and meta-analysis summarizes the individual and flock prevalence of SRLV throughout the world and describes the diagnostic tests employed over the last four decades. Our results indicate that Europe is the continent with the most information on the prevalence of the infection as well as with the highest SRLV prevalence at the individual level. Flock prevalence depends directly on the individual prevalence. SRLV diagnostic methods in sheep have substantially changed during the last decades, but serologic methods have always been the most frequently used techniques for prevalence studies. A combination of at least two diagnostic tests is fully encouraged for future prevalence studies and health programs. ELISA and PCR show synergic effects in SRLV diagnosis.

**Abstract:**

Small Ruminant Lentiviruses (SRLV) are highly prevalent retroviruses with significant genetic diversity and antigenic heterogeneity that cause a progressive wasting disease of sheep called Maedi-visna. This work provides a systematic review and meta-analysis of the last 40 years (1981–2020) of scientific publications on SRLV individual and flock prevalence. Fifty-eight publications and 314 studies were included. Most articles used a single diagnostic test to estimate prevalence (77.6%), whereas articles using three or more tests were scarce (6.9%). Serological tests are more frequently used than direct methods and ELISA has progressively replaced AGID over the last decades. SRLV infection in sheep is widespread across the world, with Europe showing the highest individual prevalence (40.9%) and being the geographical area in which most studies have been performed. Africa, Asia, and North America show values between 16.7% to 21.8% at the individual level. South and Central America show the lowest individual SRLV prevalence (1.7%). There was a strong positive correlation between individual and flock prevalence (ρ = 0.728; *p* ≤ 0.001). Despite the global importance of small ruminants, the coverage of knowledge on SRLV prevalence is patchy and inconsistent. There is a lack of a gold standard method and a defined sampling strategy among countries and continents.

## 1. Introduction

Maedi-visna is a progressive wasting disease of sheep that causes important deleterious effects in animal production and limits animal trade worldwide [1,2,3]. This condition is caused by Small Ruminant Lentiviruses (SRLV), a group of single-stranded RNA viruses with high mutation and recombination potential [4]. Indeed, five main genotypes (A–E) and more than 28 subgroups have been already characterized [5]. This phylogenetic diversity implies high genetic and antigenic diversity, which hinder serologic and molecular diagnosis [6].

SRLV have tropism for the mononuclear-phagocyte system and induce slow, chronic, and persistent inflammation in four main target organs, namely lung, joints, nervous system, and mammary gland, inducing different clinical forms (i.e., pulmonary, articular, nervous, and mammary). Interestingly, the occurrence of each clinical form as well as the severity of the lesions depend on viral factors as well as the host immune response [1,3,7,8]. The most common issue after SRLV infection is increased replacement rates due to decreased animal condition and production [1].

There are no treatments or commercial vaccines for Maedi-visna. Thus, accurate diagnosis is the cornerstone for setting up an optimal control program of the infection and reduce its prevalence. Multiple diagnostic techniques can be used to detect SRLV infection. Indirect methods (AGID and ELISA) have been proposed as the most appropriate to detect infected animals, ELISA having higher sensitivity and lower specificity than AGID [9]. Direct methods to detect SRLV (PCR, indirect immunofluorescence, and *in situ* hybridization) are also efficient diagnostic techniques [10]. Recent studies have demonstrated the inherent inaccuracy of using a single diagnostic test [11], which is likely related to the wide SRLV antigenic diversity. However, host response can also play a role since animals from the same herd infected with the same SRLV exhibit significant differences in the susceptibility to infection and viral replication [12]. Furthermore, delay in seroconversion can be very variable among individuals [13]. Although initial descriptions of SRLV infection are from the 1950s [14], only during the last forty years has there been a growing body of publications assessing individual and flock SRLV prevalence around the world. However, a comprehensive compilation of the diagnostic methods used and their prevalence results in each continent is lacking. 

The aim of this work is to estimate and compare the prevalence of SRLV in the world by performing a systematic review and meta-analysis of the articles published during the last 40 years (1981–2020) complemented by comprehensive description of diagnostic test used.

## 2. Materials and Methods

### 2.1. Literature Search and Recording of Information 

Literature of the last 40 years (1981–2020, both included) dealing with SRLV prevalence in sheep was collected following PRISMA guidelines [15,16]. A flow diagram describing the selection process of references is detailed in Appendix A. Different databases were checked including PubMed, WOS, and Scopus. Keywords included: maedi, maedi-visna, maedi/visna, small ruminant lentivirus, SRLV, and/or prevalence. Criteria for eligibility were: (i) detailed information on SRLV prevalence in countries or regions within countries; (ii) abstract written or translated in English; and (iii) publication between 1981 and 2020, inclusive. The reference year of each prevalence study was the date on which the study was performed. Exceptionally, for publications in which this date was not available, the date of article publication was used. Criteria for exclusion were: (i) total number of sampled animals not detailed; and (ii) studies focused on diseased sheep.

Data from publications included in this meta-analysis were extracted by a single researcher (M.A.) and confirmed by three different investigators (R.d.M., A.R.L., and E.P.). Data items systematically collected are detailed in Appendix B.

### 2.2. Analyses

Qualitative epidemiological variables obtained from publications (i.e., presence or absence of data on animal prevalence, flock prevalence, and population size) and information on diagnostic techniques (i.e., presence or absence of data on sensitivity and specificity and number and type of tests used) were analyzed using contingency tables and represented as absolute and relative frequencies. Additionally, the type of diagnostic tests used for SRLV prevalence determinations was described over the four decades that this meta-analysis includes. A test was considered as diagnostic when applied to all samples collected or a randomly selected group of them. Graphs were produced with Prism 8.0.2 (GraphPad Software).

The five main statistical parameters used in this meta-analysis are detailed in Table 1. Apparent prevalence (percentage of positive animals) was used as most publications did not detail specificity and sensitivity of diagnostic tests, and consequently true prevalence (percentage of infected animals) could not be estimated. When the number of positive reactors to the test was not provided in the study, it was calculated with the prevalence and the sample size. When multiple diagnostic tests were performed, animals were considered positive if they were positive to at least one diagnostic test. Data obtained from publications were subdivided into two main groups: individual and flock prevalence. Moreover, prevalence data were grouped by continents and countries and values were calculated as the weighted arithmetic mean to attribute each study its relative importance, depending on sample size. The 95% confidence interval for the estimated prevalence values was calculated by using the formula of Wilson et al. [17]. This indicator provides a range of values in which the population prevalence can be found with a 95% degree of confidence. Heterogeneity of studies was quantified with the heterogeneity statistic I^2^. This parameter is based on Cochran’s Q test of homogeneity and provides useful information in the variability between the studies included in the meta-analysis. Additionally, an historical evolution of the infection by decades was performed. All the above-mentioned analyses were performed with Excel (Microsoft Office Professional Plus 2019, USA), except for heterogeneity statistic I^2^, which was calculated with OpenMeta[Analyst] software [18]. Maps were produced with GeoNames extension (DSAT, Microsoft Office Professional Plus 2019, United States) for Excel. Finally, correlation and determination coefficients between animal and flock prevalence were calculated with Spearman’s rank test by using IBM SPSS 19.0 for Windows (IBM Corporation, Armonk, NY, USA). 

## 3. Results

### 3.1. Analysis of Publications and Diagnostic Tests

**Figure 1:**
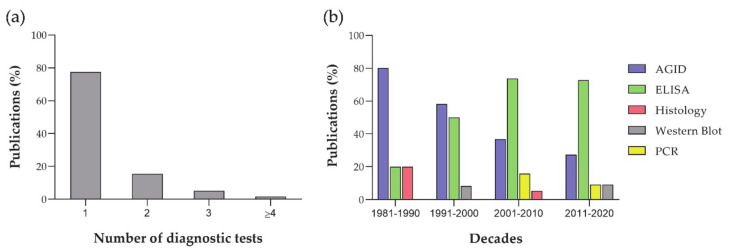
Analysis of diagnostic tests: (**a**) number of diagnostic tests performed in each article; and (**b**) evolution of the diagnostic tests from 1981 to 2020.

In total, 58 publications were included in this meta-analysis (Appendix A). All these publications showed individual prevalence studies, whereas 65.5% (38/58) also contained flock prevalence studies. In total, 314 prevalence studies were recorded. Information on the total number of animals and flocks in the studied geographical area (global population size) was only provided in 31% (18/58) and 34.2% (13/38) of publications, respectively. Sensitivity and specificity of the diagnostic tests were detailed in 36.2% (21/58) of publications. Most articles used a single diagnostic test (77.6%, 45/58) to calculate prevalence, whereas articles using three or more tests were scarce (6.9%, 4/58; Figure 1a). AGID was the most common diagnostic test from 1981 to 2000, showing a decreased importance over the years (Figure 1b). The use of ELISA showed a marked increase from 1981 to 2000, becoming the most important technique from 2001 to 2020, with constant values over the two decades. PCR has been used as a diagnostic tool for prevalence studies from 2001 to 2020, but there is a scarcity of publications using this technique as the main diagnostic tool. Histology and Western blot have been occasionally used as diagnostic tools.

### 3.2. Individual SRLV Prevalence

Prevalence of SRLV infection in sheep per continent is provided in Table 2. Europe shows by far the highest value for SRLV prevalence (40.9%), whereas Africa, Asia, and North America show values in a range between 16.7% to 21.8%. South and Central America show the lowest individual SRLV prevalence (1.7%). Interestingly, results for Europe derive from 65 studies of SRLV prevalence in 22 publications with a total of 407,509 sheep tested during the last 40 years, whereas Africa only shows five studies in foru publications and a total of 1688 animals studied during the last four decades. All continents showed marked heterogeneity among studies, with prevalence values ranging from 0% to 71.2%. Appendix C provides an evolution of individual SRLV prevalence along decades in each continent.

**Table 2:** Individual SRLV prevalence in each continent. Data extracted from scientific literature published from 1981 to 2020.

Continent	Studies	N ^1^	Prevalence (%)		Range (%)		Heterogeneity	Refs
			Mean	CI 95% ^2^	Min	Max	I^2 3^ (*p* Value)	
Africa	5	1688	16.65	14.95–18.50	1.37	24.80	98.09 (<0.001)	[19,20,21,22]
Asia	47	8309	20.38	19.52–21.26	0.00	71.20	98.60 (<0.001)	[23,24,25,26,27,28,29,30,31,32,33,34,35]
Europe	65	407,509	40.90	40.75–41.05	0.00	66.43	98.98 (<0.001)	[11,23,24,25,26,27,28,29,30,31,32,33,34,35,36,37,38,39,40,41,42,43,44,45,46,47,48,49,50,51,52,53,54,55,56]
North America	46	124,542	21.76	21.53–21.99	0.00	52.00	99.61 (<0.001)	[57,58,59,60,61,62,63,64,65,66]
South and Central America	41	46,418	1.67	1.56–1.79	0.00	30.00	91.52 (<0.001)	[67,68,69,70,71,72,73,74,75]
Global	204	588,466	33.39	33.27–33.51	0.00	71.20	99.95 (<0.001)	

^1^ N, number of animals tested; ^2^ CI 95%, 95% confidence interval; ^3^ I^2^, heterogeneity statistic.

**Figure 2:**
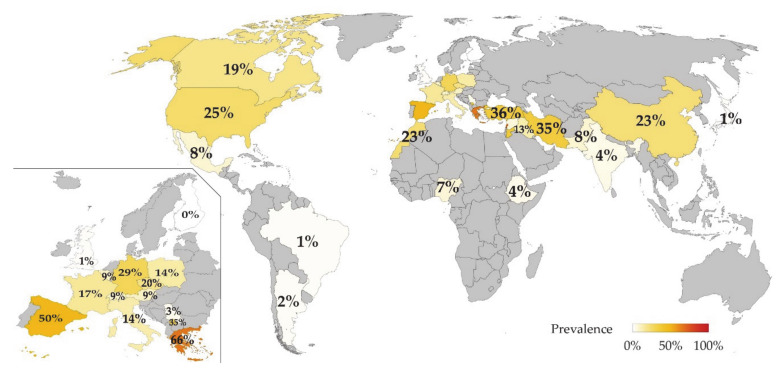
Individual SRLV prevalence (%) in sheep per country. Data extracted from scientific literature published from 1981 to 2020 and detailed in Table 2. Inset: Higher magnification of Europe.

Infected animals per country are detailed in Figure 2 and extended information is provided in Appendix D. In total, 33 countries provided studies with valid data and were located in: Africa (n = 3), Asia (n = 10), Europe (n = 14), North America (n = 3), and South and Central America (n = 3). Countries with the highest individual SRLV prevalence are Lebanon, Greece, and Spain. Spain is the country with the highest number of animals studied (308,858).

### 3.3. Flock SRLV Prevalence

Prevalence of flock SRLV infection per continent is provided in Table 3. Asia is the continent showing the highest flock prevalence (66%), whereas Europe and North America are within a range of 44.4–48.6%. Africa is the continent showing a lesser percentage of prevalence (7.7%). Flock SRLV prevalence showed marked heterogeneity among studies in all continents. Appendix E provides an evolution of SRLV prevalence along decades in each continent. Distribution of flock SRLV prevalence along decades parallels the temporal evolution of individual prevalence.

**Figure 3:**
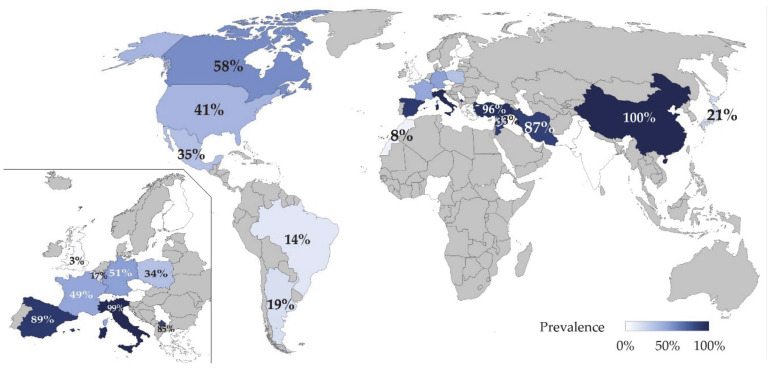
Flock prevalence (%) of SRLV in sheep by country. Data extracted from scientific literature published from 1981 to 2020 and detailed in Table 3. Inset: Higher magnification of Europe.

Infected flocks per country are detailed in Figure 3 and extended information is provided in Appendix F. In total, 23 countries provided studies with valid data and were located in: Africa (n = 1), Asia (n = 7), Europe (n = 9), North America (n = 3), and South and Central America (n = 3). Countries with the highest SRLV flock prevalence are Lebanon and China, whereas Poland is the country with the highest number of flocks studied (1621).

### 3.4. Correlation between Individual and Flock SRLV Prevalence

**Figure 4:**
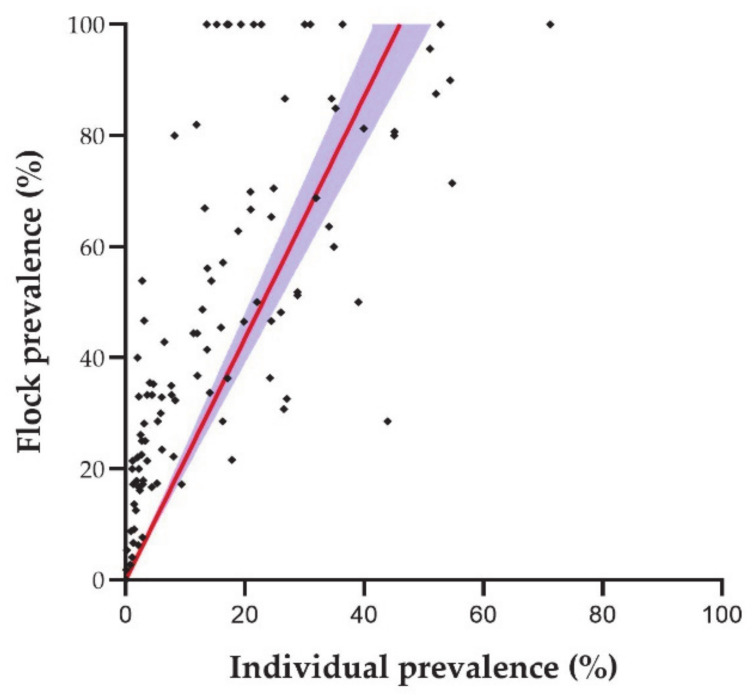
Correlation between individual and flock SRLV prevalence: dots are the data of each individual study (n = 118); the red line is the linear regression; and the blue area is the 95% confidence interval of the linear regression [11,21,25,26,28,29,31,33,35,36,37,38,41,42,45,47,48,49,51,52,54,57,58,59,61,62,63,64,65,66,67,69,71,72,73,74,75].

Individual and flock prevalence was obtained from 118 studies. There was a strong positive correlation between individual and flock prevalence (ρ = 0.728; *p* ≤ 0.001). Linear regression (y = 2.174x) demonstrated that each increase in individual prevalence induced at least two-fold increase in flock prevalence. Indeed, in some cases, flock prevalence reached 60% when individual prevalence was below 30% (Figure 4). The determination coefficient was 0.530. 

## 4. Discussion

This meta-analysis based on published research presents the distribution and prevalence of SRLV considering individual animals and flocks in the world over the last 40 years. The results indicate a widespread SRLV infection in all continents and underline the importance of SRLV in sheep throughout the world. SRLV is heterogeneously distributed, with marked variations not only between continents but also between regions in the same continent. This heterogeneity between studies reflects the multiple factor that influence SRLV prevalence.

Europe is the continent with most information on prevalence and distribution of infection as 1/3 of publications included in the study and 2/3 of animals analyzed belong to this continent. Recent phylogenetic studies suggest that SRLV-genotype A, historically associated with Maedi-visna in sheep, may have arisen in a territory within the current borders of Turkey and spread across the world with human migratory movements [25,76]. First reports of lesions compatible with those caused by SRLV were likely reported in The Netherlands [77] and the description of the disease together with the infection took place in Iceland [78]. This could explain the marked interest of European countries in the study of this infection. North America and Asia also show a notable SRLV prevalence and a growing number of published studies in both continents. There are striking differences among countries in the number of animals tested against SRLV, Spain being the country with highest number of studied animals (n = 308,858) and Pakistan the country with the fewest tested sheep (n = 93). Interpretation of prevalence studies with low sample size should be performed cautiously.

SRLV prevalence data depend on the different routes for viral spread. Horizontal (aerosols and direct contact) transmission is the main route for SRLV propagation, and it is likely the route responsible for most of the SRLV clinical cases [1]. This route is influenced by numerous environmental, demographic, and management factors [1,65]. Additionally, vertical lactogenic transmission also plays an important epidemiological role, with up to 16% of lambs born from seropositive ewes being infected during their first day of life [79]. The high individual SRLV prevalence found in Europe is likely associated with high density of ovine populations and intensive management. For instance, Greece and Spain show the highest prevalence values in Europe and both countries are within the top ten countries in milk production [80], which is usually performed in intensive management systems and implies continuous close contact within animals. However, Italy is also among this top ten countries, but its prevalence is not that high. Average flock sizes in dairy sheep are similar in Greece, Spain, and Italy (140–161 ewes/farm) [81], thus this factor cannot explain the Italian moderate SRLV prevalence values. Note that Europe is the third continent in the ranking of flock prevalence despite being the first in individual prevalence. This decrease might be associated to the several control and eradication programs performed in European flocks during the last decades.

Iran, Turkey, China, Jordan, and Lebanon are the Asiatic countries with the highest SRLV individual prevalence. Indeed, Iran, Turkey, and China are major producers of meat and milk [80,82], further highlighting the relevance of the production type and management system [83,84,85]. Based on the FAO database, Asia is the continent with the highest ovine population of the world and this fact could be determinant in SRLV transmission between flocks, as Asia is the continent with the highest flock SRLV prevalence. Interestingly, most ovine breeding stocks are located in China and India and individual SRLV prevalence in these countries is strikingly different, pointing out that the total number of animals in a given geographical area is not a relevant factor for intra-flock SRLV transmission. Differences between individual prevalence values of these two countries could be likely explained by differences in management systems, being mainly semi-intensive to intensive in the former and extensive to nomadic in the latter [80,86]. A similar explanation could be applied to the differences found between individual prevalence values in African countries, where Morocco shows much higher values than Ethiopia and Nigeria, for instance. About 75% of sheep in Ethiopia are kept on small-scale mixed farms, with an average number of 5.3 sheep per farm, usually raised in privately owned land [80,87,88]. The Animal and Plant Health Inspection Service of the United States Department of Agriculture provides an outstanding explanation on the influence of cultural, geographical and management factors in SRLV prevalence in North America [65] showing the significant role of transport (i.e., crowding of animals) and the knowledge and concern of farmers about the disease. Finally, low values of individual SRLV prevalence in South America are likely associated with the low number of animals in this region together with management factors, mostly extensive rearing [83,84,85].

The results of this meta-analysis greatly depend on the generation of prevalence data and their publication in scientific repositories. Indeed, a discrepancy between OIE reports and the information available in the scientific literature on SRLV prevalence was noted. Based on the information provided by the OIE (Disease Timelines of the World Animal Health Information Database, WAHIS interface [17]), there are 28 additional countries (Andorra, Bosnia and Herzegovina, Bulgaria, Chile, Colombia, Comoros, Croatia, Cyprus, Denmark, Estonia, North Macedonia, Hungary, Israel, Latvia, Liechtenstein, Luxembourg, Malta, Mexico, Mongolia, Netherlands, Norway, Autonomous Palestinian Territories, Portugal, Romania, Russia, Slovakia, Slovenia, and Sweden.) with SRLV infection in sheep that are absent of our study because no public publications from those countries were found, or they did not fulfilled the inclusion criteria. On the contrary, from 2005 to 2019, China, Costa Rica, Iran, Morocco, and Pakistan have never reported SRLV infection to the OIE and India, Lebanon, Czech Republic, Syria, and Turkey appear as “disease absent” despite available scientific descriptions from all these countries [20,21,23,24,25,26,27,29,30,33,34,35,55,67]. First reports on SRLV infection in Brazil according to OIE are from 2017, whereas scientific publications already reported the disease in 2007 [69]. Therefore, publicly available scientific literature might not reflect the real situation in different countries; for instance, a prevalence of 0.7% is the only datum available from the UK [42], but the infection seems to be much more widespread, reaching a high number of flocks and individual animals. Indeed, recent studies in Scottish flocks with a novel multiplex ELISA (MVD-Enferplex GSMD multiplex ELISA Kit, MV Diagnostics, Edinburgh, UK) revealed an individual prevalence of 11.7% (sample size: 2659 animals) and SRLV infection present in 15 out of 17 studied flocks (N. Watt, unpublished data, 2020). Accordingly, flocks with more than 10 years within the Scottish maedi-visna control scheme showed spontaneous outbreaks of disease with up to 90% of animals infected [89,90].

As expected, the results clearly indicate that flock prevalence is linked to individual prevalence. Indeed, flock prevalence generally doubles individual prevalence. Interestingly, some infected areas show a 100% prevalence in flocks while the infection is just about 20% in animals, suggesting that the multiplying factor could be higher than two-fold under certain conditions. Increased prevalence is related to any activity that may imply prolonged close contact between animals favoring horizontal transmission such as intensive production system [1,65], transportation, or sharing milking machines [43,91]. Flock size might also play a role as higher numbers of animals per flock relate to higher prevalence [59,61].

SRLV diagnostic methods in sheep have substantially changed during the last decades but serologic methods have always been the most used techniques in prevalence studies. AGID was the most common technique in the 1980s and 1990 as it was the recommended test by the OIE for regulatory purposes [9]. However, from the beginning of the 21st century, several ELISA tests have replaced AGID as the most reliable method. Interestingly, most of the publications included in this meta-analysis used only a single diagnostic test to estimate the SRLV prevalence. The use of a single test has proved to underestimate the number of infected animals, impairing proper segregation of infected and non-infected individuals, which leads to a slower control of the infection and hinders accurate analysis of productive and clinicopathological parameters [11,25]. In the current situation of uncertainty regarding circulating SRLV strains, the most reliable/efficient strategy to identify infected animals would involve performing at least two diagnostic tests in parallel. The election of the most suitable diagnostic tests should be carefully considered based on the most prevalent circulating strain/s in a geographical area. The first test should be ELISA-based as they are highly sensitive and specific, thus suitable for high-throughput testing [9]. However, sensitivity and specificity of any ELISA diagnostic test should not be considered universal due to the scarcity of cross-reacting antibodies among different SRLV genotypes [92,93]. Serologic approaches may have disadvantages as they cannot detect animals with low antibody titers, that can remain as carriers and potentially cause disease outbreaks [94]. The second test should be complementary to the first one and targeted towards specific animal populations. For instance, most of the publications analyzed here excluded animals younger than six months as colostral antibodies can interfere with serologic testing. This could imply overlooking an important group of animals that are pivotal for SRLV transmission [79,95]. Direct methods such as PCR could mitigate ELISA drawbacks by detecting the viral load peak found in infected lambs during the neonatal period [62,96]. Therefore, using a direct technique as a second diagnostic test can help to detect recently-infected animals without a detectable serologic response [25,97]. A combination of ELISA and PCR has already been proposed as a synergic way to accurately diagnose SRLV infection, as it provides excellent results and improves the accuracy of the diagnosis in both, acute and chronic infections [13,22,31,32,98]. Until recently, a commercial diagnostic PCR was not available commercially [11] despite several publications setting up different protocols [99]. Therefore, this strategy can increase diagnosis sensitivity and potentially imply success in SRLV control strategies. However, it could simultaneously increase costs and, potentially, reduce diagnosis specificity. In any case, this strategy has proven to be useful as it has demonstrated the infection in flocks that were previously declared as uninfected and it has improved the sensitivity of the diagnosis in countries such as Spain [11,100], UK [89], and Switzerland [101].

This analysis is based on multiple publications with important differences in the study design and efforts to group publications based on animal information (breed, sex, or age) were fruitless. Despite this heterogeneity between SRLV prevalence, the present meta-analysis provides a unique and valuable approach to worldwide SRLV distribution. Moreover, review of individual publications can help to dissect the influence of these individual parameters. For instance, breed is clearly a predisposing factor in multiple geographic areas [19,25,59]. In this sense, genetic selection of resistant animals has been proposed as a control method, with TMEM154 as a promising target gene [102]. However, a recent study demonstrates that control measures based on a single gene may not be as useful as expected [100]. Age also seems to be related to higher prevalence of infection, with a non-linear increase that reaches the maximum at about four years of age [59,61,65]. Influence of sex in prevalence is not that obvious, some studies indicate males being more predisposed than females [43], whereas others indicate non-significant differences [57,59,63].

Limitations of this work arise from the diversity of study designs and data expression. Specificity and sensitivity of the test was only specified in 36.2% of publications and we decided to deal with apparent prevalence to avoid disregarding most of the selected publications. Technical information of the diagnostic test employed should be stated whenever possible to ease further data analysis [103,104]. Further studies investigating the sensitivities and specificities of the test that were not provided in the studied publications will be useful and interesting so that further analysis of these data based on real prevalence values could be performed. Unfortunately, recurring prevalence studies in the same geographical area are scarce. A publication bias might exist when SRLV infection is discovered in a certain area, likely leading to an increased number of reports, paralleling the increase of scientific interest. In areas where the disease is enzootic, the interest might not be similar.

## 5. Conclusions

SRLV infection in sheep is widespread across the world, Europe showing the highest individual prevalence and being the geographical area in which more studies have been performed. Prevalence of infected flocks shows a strong correlation with the individual prevalence. Most studies are based on a single diagnostic test, implying a risk of underestimating the real infection prevalence. Serological tests are more commonly used than direct methods and, among them, ELISA has progressively replaced AGID along the last two decades. There is a moderate disagreement between the information reported to the OIE and the scientific literature. This review highlights the need for more systematic and frequent prevalence studies using a consistent testing strategy.

## Figures and Tables

**Table 1:** Definition of the parameters used in the epidemiological meta-analysis.

Mathematical Equations
Apparent prevalence = (Pos/n)∙100
Positive animals or flocks = (P∙n)/100
Confidence interval = (2nP + Z_(α/2)^2 ± Z_(α/2) √(4nP(1 − P) + Z_(α/2)^2))/(2(n + Z(α/2)^2))
Weighted arithmetic mean of prevalence = (∑ (Pi∙ni))/(∑ni)
Heterogeneity statistic I^2^ = ((Q − df)/Q) × 100

Pos, number of positive animals; n, sample size; P, prevalence value; Z value, varies depending on the percent of confidence; α, alpha level or difference between 100% and the confidence interval; Pi, prevalence value of each study to be averaged; ni, sample size of each study to be averaged; Q, Cochran’s homogeneity test statistic; df, degrees of freedom.

**Table 3:** Flock SRLV prevalence in each continent. Data extracted from scientific literature published from 1981 to 2020.

Continent	Studies	N ^1^	Prevalence (%)		Range (%)		Heterogeneity	Refs
			Mean	CI 95% ^2^	Min	Max	I^2 3^ (*p* Value)	
Africa	1	13	7.69	1.37–33.31	7.69	7.69	N/A ^4^	[21]
Asia	8	197	65.99	59.12–72.24	21.43	100.00	95.58 (<0.001)	^5^
Europe	35	4590	44.38	42.95–45.82	0.00	100.00	99.98 (<0.001)	^6^
North America	32	1933	48.58	46.35–50.81	0.00	100.00	95.26 (<0.001)	^7^
South and Central America	34	2358	18.87	17.34–20.50	0.00	100.00	83.15 (<0.001)	^8^
Global	110	9091	39.07	38.07. 40.08	0	100	99.94 (<0.001)	

^1^ N, number of animals tested; ^2^ CI 95%, 95% confidence interval; ^3^ I^2^, heterogeneity statistic; ^4^ N/A, not applicable; ^5^ [25,26,28,29,31,33,35]; ^6^ [11,36,37,38,39,40,41,42,45,47,48,49,51,54,55]; ^7^ [57,58,59,61,62,63,64,65,66]; ^8^ [67,69,71,72,73,74,75].

**Figure A1:**
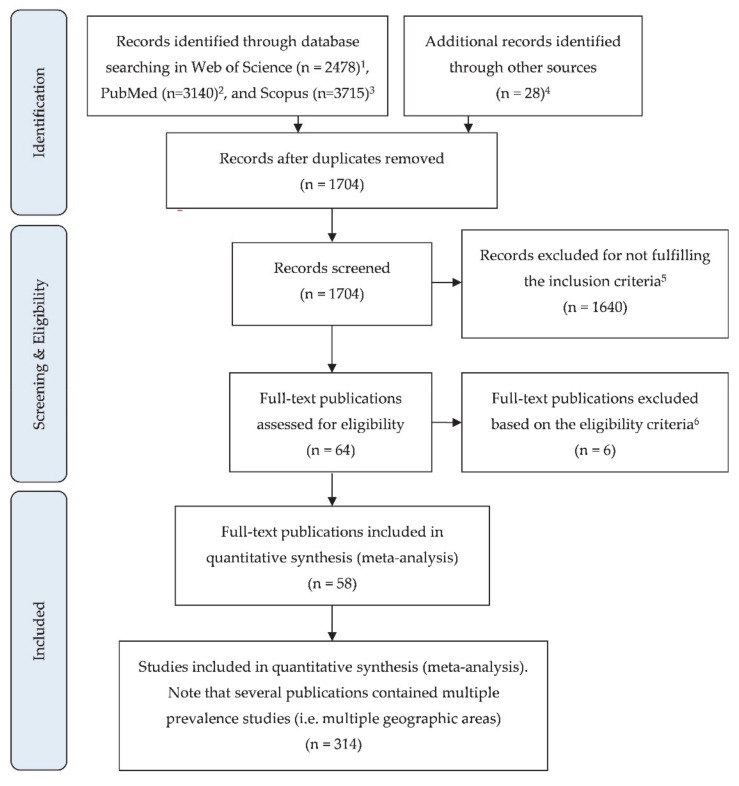
1 Keywords in Web of Science. maedi: 1641; maedi prevalence: 196; maedi-visna: 161; maedi-visna prevalence: 161; maedi/visna: 161; SRLV: 40; SRLV prevalence: 40; small ruminant lentivirus: 39; small ruminant lentivirus prevalence: 39. 2 Keywords in Pubmed. maedi: 733; maedi prevalence: 156; maedi-visna: 709; maedi-visna prevalence: 152; maedi/visna: 709; SRLV: 189; SRLV prevalence: 73; small ruminant lentivirus: 328; small ruminant lentivirus prevalence: 91. 3 Keywords in Scopus. maedi: 1019; maedi prevalence: 189; maedi-visna: 578; maedi-visna prevalence: 150; maedi/visna: 968; SRLV: 281; SRLV prevalence: 85; small ruminant lentivirus: 326; small ruminant lentivirus prevalence: 119. 4 Other sources. Conference Proceedings: 9; Google Scholar: 19. 5 Eligibility criteria: Information on SRLV prevalence; abstract available in English; publication from 1981 to 2020, inclusive. 6 Exclusion criteria: Total number of sampled animals not detailed; study focused on diseased sheep.

## Data Availability

The data presented in this study are available in the scientific publications listed in the Reference section.

## Appendix A

Flow diagram indicating procedure for identification, screening, selection and inclusion of publications in this meta-analysis. Modified from the PRISMA Statement [93].

## Appendix B

The following is the information that was systematically extracted from the publications included in the meta-analysis.

- Information related to the publication time and location:
  ◦ Country of the study
  ◦ Continent of the study
  ◦ Year of the study
  ◦ Year of the publication
- Information related to diagnostic procedures:
  ◦ Number of techniques employed in the study
  ◦ Type of diagnostic technique
  ◦ Sensitivity and specificity of tests
- Information related to epidemiological data of the population:
  ◦ Total number of animals in the geographical area of the study
  ◦ Total number of flocks in the geographical area of the study
- Information related to epidemiological data of the sample:
  ◦ Sample size
  ◦ Number of positive animals and/or flocks (A flock was considered positive when one or more animals were positive to SRLV test)
  ◦ Subgroups of samples within the same publication. (i.e., prevalence in different regions, which were considered as independent studies)
- Information related to production system factors:
  ◦ Flock size
  ◦ Type of management system
- Information related to the animal factors:
  ◦ Breed of sampled animals
  ◦ Sex of sampled animals
  ◦ Age of sampled animals

## Appendix C

Individual SRLV prevalence in each continent by decades. Mean, number of animals tested (N), 95% Confidence Interval (CI 95%), and range are provided.

**Table A1:** Animal SLRV prevalence in each continent from 1981 to 1990.

Continent	Studies	N	Prevalence		Range	
			Mean	CI 95%	Min	Max
Africa	1	72	2.78	0.77–9.57	2.78	2.78
Asia	0	0	N/A ^1^	N/A	N/A	N/A
Europe	5	24,969	16.34	15.88–16.80	1.70	26.67
North America	19	57,593	19.13	18.81–19.45	0.00	39.92
South and Central America	0	0				
Global	25	82,634	18.27	18.01–18.54	0.00	39.92

^1^ N/A, not applicable.

**Table A2:** Animal SLRV prevalence in each continent from 1991 to 2000.

Continent	Studies	N	Prevalence		Range	
			Mean	CI 95%	Min	Max
Africa	2	1267	20.99	18.84–23.32	6.74	24.80
Asia	13	2890	6.02	5.21–6.95	0.00	12.20
Europe	11	26,232	9.10	8.76–9.45	0.00	24.00
North America	21	39,444	26.49	26.05–26.92	2.19	52.00
South and Central America	0	0	N/A ^1^	N/A	N/A	N/A
Global	47	69,833	19.01	18.72–19.30	0.00	52.00

^1^ N/A, not applicable.

**Table A3:** Animal SLRV prevalence in each continent from 2001 to 2010.

Continent	Studies	N	Prevalence		Range	
			Mean	CI 95%	Min	Max
Africa	2	349	3.72	2.19–6.27	1.37	5.42
Asia	17	3889	28.57	27.17–30.01	0.00	68.97
Europe	5	296,695	51.25	51.07–51.43	24.80	66.43
North America	3	23,923	22.21	21.69–22.74	1.95	28.82
South and Central America	18	26,529	1.09	0.97–1.22	0.00	4.42
Global	45	351,385	45.19	45.02–45.35	0.00	68.97

**Table A4:** Animal SLRV prevalence in each continent from 2011 to 2020.

Continent	Studies	N	Prevalence		Range	
			Mean	CI 95%	Min	Max
Africa	0	0	N/A ^1^	N/A	N/A	N/A
Asia	17	1530	26.67	24.51–28.94	4.29	71.20
Europe	44	59,613	13.65	13.38–13.93	0.00	54.73
North America	3	3582	8.93	8.04–9.91	4.63	15.19
South and Central America	23	19,889	2.45	2.24–2.67	0.00	30.00
Global	87	84,614	11.05	10.84–11.27	0.00	71.20

^1^ N/A, not applicable.

## Appendix D

**Table A5:** Individual SRLV prevalence in each country. Mean, 95% confidence interval, and range are provided. Data extracted from scientific literature published from 1981 to 2020.

Country	Studies	N ^1^	Prevalence (%)		Range (%)		Refs
			Mean	CI 95% ^2^	Min	Max	
AFRICA							
Ethiopia	2	349	3.72	2.19–6.27	1.37	5.42	[19]
Morocco	2	1072	23.32	20.89–25.94	2.78	24.80	[20,21]
Nigeria	1	267	6.74	4.31–10.40	6.74	6.74	[22]
ASIA							
China	12	672	22.77	19.76–26.09	5.36	50.00	[29]
India	1	140	4.29	1.98–9.03	4.29	4.29	[24]
Iraq	1	210	12.86	8.99–18.06	12.86	12.86	[32]
Iran	1	220	34.55	28.58–41.05	34.55	34.55	[26]
Japan	4	771	0.78	0.36–1.69	0.00	1.24	[31]
Jordan	1	231	36.36	30.43–42.74	36.36	36.36	[28]
Lebanon	1	184	71.20	64.27–77.25	71.20	71.20	[33]
Pakistan	1	93	7.53	3.69–14.73	7.53	7.53	[34]
Syria	13	2890	6.02	5.21–6.95	0.00	12.20	[35]
Turkey	12	2898	35.51	33.79–37.27	0.00	68.97	[23,25,27,30]
EUROPE							
Austria	1	883	9.40	7.65–11.50	9.40	9.40	[56]
Belgium	1	555	9.37	7.22–12.08	9.37	9.37	[54]
Czech Republic	1	2801	19.85	18.41–21.37	19.85	19.85	[55]
Finland	1	10,802	0.00	0.00–0.04	0.00	0.00	[45]
France	3	23,404	16.68	16.20–17.16	1.70	26.67	[41]
Germany	2	2252	28.51	26.68–30.41	0.00	28.80	[46,51]
Greece	1	143	66.43	58.35–73.65	66.43	66.43	[53]
Italy	1	682	13.64	11.26–16.42	13.64	13.64	[49]
Kosovo	6	10,544	35.19	34.28–36.10	12.92	45.93	[48]
Poland	16	19,253	14.33	13.84–14.83	0.16	54.73	[39,52]
Serbia	14	11,709	3.38	3.07–3.73	0.00	9.96	[43]
Spain	16	308,858	49.84	49.67–50.02	1.23	54.41	[11,36,37,38,40,47,50]
Switzerland	1	3866	9.00	8.14–9.94	9.00	9.00	[44]
United Kingdom	1	11,757	0.74	0.60–0.91	0.74	0.74	[42]
NORTH AMERICA							
Canada	29	68,019	19.21	18.92–19.51	0.00	50.00	[57,60,62,63,66]
Mexico	1	157	7.64	4.43–12.88	7.64	7.64	[64]
United States	16	56,366	24.87	24.51–25.23	2.19	52.00	[58,59,61,65]
SOUTH AND CENTRAL AMERICA							
Argentina	29	42,597	1.69	1.57–1.82	0.00	30.00	[72,73]
Brazil	6	3103	1.32	0.98–1.79	0.11	8.20	[68,69,70,71,74,75]
Cosa Rica	6	718	1.95	1.16–3.25	0.00	4.26	[67]

^1^ N, number of animals tested. ^2^ CI 95%, 95% confidence interval.

## Appendix E

Flock SRLV prevalence in each continent by decades. Mean, number of flocks tested (N), 95% Confidence Interval (CI 95%), and range are provided.

**Table A6:** Flock SLRV prevalence in each continent from 1981 to 1990.

Continent	Studies	N	Prevalence		Range	
			Mean	CI 95%	Min	Max
Africa	1	13	7.69	1.37–33.31	7.69	7.69
Asia	0	0	N/A ^1^	N/A	N/A	N/A
Europe	4	477	58.49	54.02–62.83	12.50	98.94
North America	12	675	62.96	59.26–66.52	0.00	100.00
South and Central America	0	0	N/A	N/A	N/A	N/A
Global	17	1165	60.52	57.68–63.28	0	100

^1^ N/A, not applicable.

**Table A7:** Flock SLRV prevalence in each continent from 1991 to 2000.

Continent	Studies	N	Prevalence		Range	
			Mean	CI 95%	Min	Max
Africa	0	0	N/A ^1^	N/A	N/A	N/A
Asia	1	73	32.88	23.19–44.27	32.88	32.88
Europe	8	445	11.01	8.43–14.26	0.00	100.00
North America	15	357	53.22	48.04–58.34	20.00	100.00
South and Central America	0	0	N/A	N/A	N/A	N/A
Global	24	875	30.06	27.11–33.18	0.00	100.00

^1^ N/A, not applicable.

**Table A8:** Flock SLRV prevalence in each continent from 2001 to 2010.

Continent	Studies	N	Prevalence		Range	
			Mean	CI 95%	Min	Max
Africa	0	0	N/A ^1^	N/A	N/A	N/A
Asia	3	67	76.12	64.67–84.73	21.43	95.65
Europe	4	912	92.65	90.78–94.17	51.22	100.00
North America	2	759	34.91	31.61–38.37	22.08	36.36
South and Central America	17	1234	12.97	11.21–14.96	0.00	25.00
Global	26	2972	44.82	42.67–46.24	0.00	100.00

^1^ N/A, not applicable.

**Table A9:** Flock SLRV prevalence in each continent from 2011 to 2020.

Continent	Studies	N	Prevalence		Range	
			Mean	CI 95%	Min	Max
Africa	0	0	N/A ^1^	N/A	N/A	N/A
Asia	4	57	96.49	88.08–99.03	83.33	100.00
Europe	19	2756	31.35	29.64–33.11	2.75	100.00
North America	3	142	41.55	33.77–49.77	35.00	51.85
South and Central America	17	1124	25.36	22.90–27.98	0.00	100.00
Global	43	4079	30.96	29.56–32.40	0.00	100.00

^1^ N/A, not applicable.

## Appendix F

**Table A10:** Flock SRLV prevalence in each country. Mean, 95% confidence interval, and range are provided. Data extracted from scientific literature published from 1981 to 2020.

Country	Studies	N ^1^	Prevalence (%)		Range (%)		Refs
			Mean	CI 95% ^2^	Min	Max	
AFRICA							
Ethiopia	0	0	N/A ^3^	N/A	N/A	N/A	N/A
Morocco	1	13	7.69	1.37–33.31	7.69	7.69	[21]
Nigeria	0	0	N/A	N/A	N/A	N/A	N/A
ASIA							
China	1	24	100.00	86.20–100.00	100.00	100.00	[29]
India	0	0	N/A	N/A	N/A	N/A	N/A
Iraq	0	0	N/A	N/A	N/A	N/A	N/A
Iran	1	30	86.67	70.32–94.69	86.67	86.67	[26]
Japan	1	14	21.43	7.57–47.59	21.43	21.43	[31]
Jordan	2	17	88.24	65.66–96.71	83.33	100.00	[28]
Lebanon	1	16	100.00	80.64–100.00	100.00	100.00	[33]
Pakistan	0	0	N/A	N/A	N/A	N/A	N/A
Syria	1	73	32.88	23.19–44.27	32.88	32.88	[35]
Turkey	1	23	95.65	79.01–99.23	95.65	95.65	[25]
EUROPE							
Austria	0	0	N/A	N/A	N/A	N/A	N/A
Belgium	1	87	17.24	10.74–26.52	17.24	17.24	[54]
Czech Republic	0	0	N/A	N/A	N/A	N/A	N/A
Finland	1	340	0.00	0.00–1.12	0.00	0.00	[45]
France	3	383	48.56	43.60–53.56	12.50	86.67	[41]
Germany	1	41	51.22	36.48–65.75	51.22	51.22	[51]
Greece	0	0	N/A	N/A	N/A	N/A	N/A
Italy	1	94	98.94	94.22–99.81	98.94	98.94	[49]
Kosovo	1	318	84.91	80.56–88.42	84.91	84.91	[48]
Poland	15	1621	34.24	31.97–36.58	3.77	71.43	[39]
Serbia	0	0	N/A	N/A	N/A	N/A	N/A
Spain	11	980	89.49	87.41–91.26	6.67	100.00	[11,36,37,38,40,47]
Switzerland	0	0	N/A	N/A	N/A	N/A	N/A
United Kingdom	1	726	2.75	1.79–4.22	2.75	2.75	[79]
NORTH AMERICA							
Canada	15	849	58.30	54.96–61.58	0.00	100.00	[57,62,63,66]
Mexico	1	20	35.00	18.12–56.71	35.00	35.00	[64]
United States	16	1064	41.07	38.15–44.05	20.00	87.50	[58,59,61,65]
SOUTH AND CENTRAL AMERICA							
Argentina	29	2239	18.94	17.37–20.61	0.00	100.00	[72,73]
Brazil	4	104	14.42	8.94–22.44	1.85	80.00	[69,71,74,75]
Cosa Rica	1	15	40.00	19.82–64.25	40.00	40.00	[67]

^1^ N, number of animals tested. ^2^ CI 95%, 95% confidence interval. ^3^ N/A, not applicable.
